# Quantitative medical cost-effectiveness analysis of molecular-targeting cancer drugs in Japan

**DOI:** 10.1186/2008-2231-21-40

**Published:** 2013-05-25

**Authors:** Takeshi Ebara, Tatsuya Ohno, Takashi Nakano

**Affiliations:** 1 Department of Radiation Oncology, Saitama Medical University International Medical Center, 1397-1 Yamane Hidaka, Saitama, 350-1298, Japan; 2 Department of Radiation Oncology, Gunma University, Graduate School of Medicine, 3-39-22, Showa-machi, Maebashi, Gunma, 371-8511, Japan

**Keywords:** Cost-effectiveness analysis, Molecular-targeting drugs, Simple quantitative calculation, Japan

## Abstract

**Background:**

In Japan, both incidence and mortality rates of cancers have continuously increased and medical costs are growing more rapidly than the overall economy of Japan. However, there is no consensus threshold for cost-effectiveness in medical care, and few studies have investigated cost-effectiveness of medical care in Japan. The present study was to determine the direct costs of molecular-targeting drugs that were recently approved in Japan through simple and quantitative calculations. Thus, we calculated an incremental cost-effectiveness ratio (ICER) and the cost per life-year gained (LYG) by using reported data from randomized clinical trials for various cancers.

**Methods:**

Between 2008 and 2011, we reviewed seven molecular-targeting drugs that were approved for treatment of five cancers in Japan. These drugs included Bevacizumab, sorafenib, sunitinib, temsirolimus, Lapatinib, and panitumumab. Direct cost, ICER, and LYG of the drugs were estimated from the randomized phase III clinical trial data referred to in package leaflets. Effectiveness was defined as the prolongation of both median overall survival (OS) and progression-free survival (PFS). Costs were calculated as those of molecular-targeting drugs. Subsequently, ICER was based on 1-month increases in both OS and PFS periods and 1% increases in OS, and LYG was determined.

**Results:**

Direct costs ranged from ¥724,804 ($9,060) to ¥1,506,628 ($18,833). ICERs of the drugs ranged from ¥724,804 ($9,060) to ¥1,506,628 ($18,833) for a 1-month increase in OS. For each month of PFS, ICERs ranged from ¥372,243 ($4,653) to ¥7,399,877 ($92,498). The costs of Bevacizumab and sorafenib for treatment of HCC per 1% increase in OS were ¥376,657 ($4,708) and ¥313,733 ($3,922), respectively. LYG ranged from ¥8,697,650 ($108,721) to ¥18,079,530 ($225,994).

**Conclusions:**

Some molecular-targeting drugs are not cost-effective. Considering ethical and moral issues, we should establish economic endpoints to approve new drugs in Japan.

## Background

In Japan, both incidence and mortality rates of cancers have continuously increased since the 1960s, with more than 357,000 cancer fatalities in 2011 [1]. The Ministry of Health, Labour and Welfare approves new anticancer drugs depending on the results of clinical trials. Medical costs are growing more rapidly than the overall economy of Japan. Japanese medical costs in 2010 came to ¥37,400 billion ($468 billion), ¥3,500 billion ($44 billion) of which was spent on cancer [2]. With increasing numbers of cancer patients, medical costs have continued to increase over recent years. However, there is no consensus threshold for cost-effectiveness in medical care, and few studies have investigated cost-effectiveness of medical care in Japan.

The primary objective of the present study was to determine the direct costs of molecular-targeting drugs that were recently approved in Japan through simple and quantitative calculations. Thus, we calculated an incremental cost-effectiveness ratio (ICER) and the cost per life-year gained (LYG) by using reported data from randomized clinical trials for various cancers.

## Methods

Between 2008 and 2011, we reviewed seven molecular-targeting drugs that were approved for treatment of five cancers in Japan. These included Bevacizumab for non-squamous-cell and non-small-cell lung cancer (NSCLC), everolimus for metastatic renal cell carcinoma (RCC), sorafenib for advanced clear-cell RCC and hepatocellular carcinoma (HCC), sunitinib or temsirolimus for metastatic RCC, Lapatinib for locally advanced or metastatic breast cancer, and panitumumab for metastatic colorectal cancer. ICER and LYG of the drugs were compared using clinical data from randomized phase III clinical trials, which were referred to in package leaflets [3]. Medical effectiveness was defined as the prolongation of both median overall survival (OS) and progression-free survival (PFS). Medical costs were calculated from the costs of molecular-targeting drugs and the median or mean duration of treatments. In reports that did not present median or mean treatment duration, it was assumed that treatments continued until the occurrence of disease progression, and treatment duration was estimated accordingly. For simplicity, we assumed that average drug-dosing regimens were those appropriate for a 60-year-old male or female patient because the incidence of cancer is higher around this age range. Based on a report by the National Health and Nutrition Survey, body surface areas of male and female patients calculated from heights and weights of 165 cm and 61 kg and 152 cm and 54 kg were 1.7 m^2^ and 1.5 m^2^, respectively [4]. Drug costs were calculated for the year 2012 based on the medical fee given in the National Health Insurance drug price list [5]. Other direct and indirect costs were not included in this analysis. Subsequently, ICER, which is expressed as additional costs of new treatment per gain in clinical outcome, was calculated as follows.

ICER = Cost (A) - Cost (B)/effectiveness (A) - effectiveness.

(B) ICER was based on 1-month increases in both OS and PFS periods and 1% increases in OS. LYG was also calculated. US dollar costs were converted to Japanese Yen by multiplying by 80.

## Findings

Among trials of the seven drugs, some compared molecular-targeting drugs with placebo or best supportive care (BSC), while others compared molecular-targeting drugs with previously established drugs. Representative phase III trials are summarized in Table 1. Trial 1 determined whether addition of Bevacizumab to paclitaxel and carboplatin improved survival of patients with metastatic non-squamous-cell and NSCLC [6]. Trials 2–4 evaluated everolimus for metastatic RCC and sorafenib for advanced clear-cell RCC and HCC and made comparisons with placebo-treated patients [7-9]. Trials 5 and 6 compared the efficacy of sunitinib or temsirolimus for metastatic RCC with that of interferon alpha [10,11]. Trial 7 determined whether addition of Lapatinib to capecitabine improved survival of patients with HER2-positive, locally advanced, or metastatic breast cancers [12], and panitumumab was compared with BSC in patients with metastatic colorectal cancer [13]. Table 2 shows drug costs estimated by published studies. The direct costs of Bevacizumab, everolimus, sorafenib for RCC, sorafenib for HCC, sunitinib, temsirolimus, Lapatinib, and panitumumab were ¥3,013,255 ($37,666); ¥2,415,109 ($30,189); ¥3,581,292 ($44,766); ¥3,451,063 ($43,138); ¥6,153,336 ($76,917); ¥3,136,794 ($39,210); ¥1,787,170 ($22,340); and ¥1,151,092 ($14,389), respectively. OS and PFS data from the trials are summarized in Table 3.

In studies of Bevacizumab and Lapatinib [6,12], cost differences (cost (A) - cost (B)) were presumed to be equivalent to the cost of Bevacizumab and Lapatinib because these studies estimated additional effect of these drugs to control arm. When control arms were placebo or BSC [7-9,13], the cost (B) of the formulation was set at zero. In contrast, cost (B) in trials of sunitinib and temsirolimus [10,11] reflected the cost of interferon alpha, which was calculated in the same way as that for

**Table 1:** Drugs and details of clinical trials

**No.**	**Drug (Brand name)**	**Disease**	**Regimen**	**Control arm**	**Authors**
1	Bevacizumab (Avastin)	Non-SQ, NSCLC #	Paclitaxel and carboplatin plus Bevacizumab	Paclitaxel and carboplatin	Sandler et al. [6]
2	Everolimus (Afinitor)	Renal cell carcinoma	Everolimus	Placebo	Motzer et al. [7]
3	Sorafenib (Nexavar)	Renal cell carcinoma	Sorafenib	Placebo	Escudier et al. [8]
4	Sorafenib (Nexavar)	Hepatocellular carcinoma	Sorafenib	Placebo	Llover et al. [9]
5	Sunitinib (Sutent)	Renal cell carcinoma	Sunitinib	Interferon alpha	Motzer et al. [10]
6	Temsirolimus (Torisel)	Renal cell carcinoma	Temsirolimus	Interferon alpha	Kwitkowski et al. [11]
7	Lapatinib (Tykerb)	Breast carcinoma	Capecitabine plus Lapatinib	Capecitabine	Geyer et al. [12]
8	Panitumumab (Vectibix)	Colorectal carcinoma	Panitumumab	BSC*	Cutsem et al. [13]

#; non-squamous-cell, non–small-cell lung cancer.

*; best supportive care.

**Table 2:** Estimated drug costs

**No.**	**Drug**	**Regimen**	**Dose #**	**Cost per cycle/day**	**Median or mean cycles/days**	**Total cost**
1	Bevacizumab	15 mg/kg every 3 weeks	900 mg every 3 weeks	¥430,465 ($5,381)	7 cycles	¥3,013,255 ($37,666)
2	Everolimus	10 mg daily	10 mg = 2 tablets	¥25,422 ($328)	95 days	¥2,415,109 ($30,189)
3	Sorafenib*	800 mg daily	800 mg = 4 tablets	¥21,705 ($271)	5.5 months ##	¥3,581,292 ($44,766)
4	Sorafenib**	800 mg daily	800 mg = 4 tablets	¥21,705 ($271)	5.3 months	¥3,451,063 ($43,138)
5	Sunitinib	50 mg daily on days 1–28 of a 42-day cycle	50 mg = 4 capsules	¥34,185 ($427)	6.0 months	¥6,153,336 ($76,917)
6	Temsirolimus	25 mg weekly	25 mg = 1 vial	¥132,915 ($1,661)	5.5 months ##	¥3,136,794 ($39,210)
7	Lapatinib	1250 mg daily on days 1–14 of a 21-day cycle	1250 mg = 5 tablets	¥8,100($101)	11 cycles ##	¥1,787,170 ($22,340)
8	Panitumumab	6 mg/kg on every 2 weeks	360 mg every 2 weeks	¥287,773 ($3,597)	4 cycles	¥1,151,092 ($14,389)

#; A 60-year-old male or female patient was considered. The detail was described in the text.

##; Median or mean cycles/days was not mentioned in the literatures. Therefore, these durations were assumed from the progression-free survival period because the treatment continued until the occurrence of disease progression, unacceptable adverse events, or withdrawal of consent.

*; for renal cell carcinoma.

**; for hepatocellular carcinoma.

 the molecular-targeting drug. With improved OS of 1 month, ICERs of Bevacizumab, sorafenib for HCC, and temsirolimus were ¥1,506,628 ($18,833); ¥1,053,321 ($13,167); ¥1,232,523 ($15,407); and ¥724,804 ($9,060), respectively. ICERs of Bevacizumab and sorafenib for HCC were ¥376,657 ($4,708) and ¥313,733 ($3,922) per 1% increase in OS. For each month of PFS, ICERs of Bevacizumab, everolimus, sorafenib for RCC or HCC, sunitinib, temsirolimus, Lapatinib, and panitumumab were ¥1,772,503 ($225,156); ¥1,150,052 ($14,376); ¥1,326,404 ($16,580); ¥915,546 ($11,444); ¥1,087,206 ($13,590); ¥372,243 ($4,653); and ¥7,399,877 ($92,498), respectively. LYGs with Bevacizumab, sorafenib for HCC, and temsirolimus were ¥18,079,530 ($225,994); ¥12,639,854 ($157,998); and ¥14,790,271 ($184,878)–¥8,697,650 ($108,720), respectively (Table 4).

**Table 3:** OS and PFS in the trials

**No**	**Drug**	**Median OS (months)**		**Median PFS (months)**	
		**Control arm**	**Test arm**	**Control arm**	**Test arm**
1	Bevacizumab	10.3 15% *	12.3 23% *	4.5	6.2
2	Everolimus	#	#	1.9	4.0
3	Sorafenib+	15.9	19.3	2.8	5.5
4	Sorafenib++	7.9 33%**	10.7 44%**	4.9##	4.1##
5	Sunitinib	#	#	5.0	11.0
6	Temsirolimus	7.3	10.9	3.1	5.5
7	Lapatinib	#	#	4.4	8.4
8	Panitumumab	#	#	7.3	8.0

#; Not mentioned in the literatures.

##; The median time to symptomatic progression.

*; at 2 years.

**; at 1 year.

+; for renal cell carcinoma.

++; for hepatocellular carcinoma.

## Discussion

Substantial increase in the cost of cancer care is a global concern. The American Society of Clinical Oncology published guidelines that can be used to assess the costs of high quality cancer treatment [14]. Cost-effectiveness analyses compare ratios of incremental cost and incremental effectiveness of various strategies [15]. Quality-adjusted life years (QALYs) are commonly used to estimate the cost–utility of medical care. In the United States and United Kingdom, $50,000–100,000 and £20,000–30,000 per QALY are considered acceptable thresholds for cost-effectiveness [16]. A Japanese study on willingness to pay for one additional QALY suggests that ¥5,000,000 ($62,500) is an appropriate threshold [16]. However, few investigations examine medical cost-effectiveness, and consensus thresholds have not been established in Japan.

Determination of thresholds for cost-effectiveness in medical care is very difficult. Fojo et al. suggested that research studies with the ability to detect survival advantages of 2 months or less should only test interventions that cost less than $20,000 per course of treatment [17]. The present study shows that most molecular-targeting drug regimens cost more than ¥1,000,000 ($12,500) per 1-month gain in survival. Hence, these drugs have poor cost-effectiveness according to Fojo’s standard.

Among cancer treatments, radiation therapy has the greatest potential for cost-effectiveness in Japan [18]. The cost-effectiveness of carbon ion radiotherapy (CIRT) and conventional multimodal therapies was compared in Japanese patients with locally recurrent rectal cancers [19]. ICER per 1% increase in survival for CIRT was ¥6,428 ($80), much cheaper than that for Bevacizumab and sorafenib (¥376,657 and ¥313,733, respectively).

Molecular-targeting drugs offer novel and attractive strategies for improving patient survival and quality of

**Table 4:** **Incremental cost**-**effective ratio**

**No.**	**Drug**	**Cost (A) - cost (B)#**	**Increase in OS (months)**	**Increase in OS (months)**	**Increase in PFS (months)**	**ICER per 1 month increase in OS**	**ICER per 1% increase in OS**	**ICER per 1 month increase in PFS**	**LYG**
1	Bevacizumab	¥3,013,255 ($37,666)	2.0	8% at 2 years	1.7	¥1,506,628 ($18,833)	¥376,657 ($4,708)	¥1,772,503 ($22,156)	¥18,079,530 ($225,994)
2	Everolimus	¥2,415,109 ($30,189)	##		2.1			¥1,150,052 ($14,376)	
3	Sorafenib+	¥3,581,292 ($44,766)	3.4		2.7	¥1,053,321 ($13,167)		¥1,326,404 ($16,580)	¥12,639,854 ($157,998)
4	Sorafenib++	¥3,451,063 ($43,138)	2.8	11% at 1 year	-0.8	¥1,232,523 ($15,407)	¥313,733 ($3,922)	Dominated	¥14,790,271 ($184,878)
5	Sunitinib	¥5,493,276 ($68,666)	*		6.0			¥915,546 ($11,444)	
6	Temsirolimus	¥2,609,295 ($32,616)	3.6		2.4	¥724,804 ($9,060)		¥1,087,206 ($13,590)	¥8,697,650 ($108,720)
7	Lapatinib	¥1,787,170 ($22,340)	**		4.0			¥372,243 ($4,653)	
8	Panitumumab	¥1,151,092 ($14,389)	***		0.2			¥7,399,877 ($92,498)	

OS; overall survival, PFS; progression-free survival, ICER; incremental cost-effective ratio, LYG; cost per life-year gained.

#; See the formulation (*) in Materials and methods and Results.

##; At the time of the analysis, median overall survival had not been reached for the everolimus group. There was no significant difference between the groups in terms of overall survival.

*; At the time of the analysis, median overall survival was not reached in either group.

**; Thirty-six deaths occurred in the Lapatinib group and 35 occurred in the control group (P = 0.72). OS curves in the literature seem to indicate no definite difference.

***; The literature mentioned that no difference was observed in OS.

+; for renal cell carcinoma.

++; for hepatocellular carcinoma.

 life after treatments. For example, Imatinib, a selective inhibitor of BCR-ABL tyrosine kinase, produces high response rates in patients with chronic-phase chronic myeloid leukemia and is considered a standard first line treatment [20]. On the other hand, molecular-targeting drugs are often only effective in a subset of patients. For example, anti-epidermal growth factor receptor antibodies should not be administered to colorectal carcinoma patients with KRAS mutations in codons 12 or 13 [21], although improved cost-effectiveness can be demonstrated for the remaining subset of patients.

Cost containment in oncology is a moral issue, and cost-effectiveness analyses are often recommended for determining how to best allocate resources. However, cost-effectiveness analyses often make prescriptions that are at odds with a sense of justice. That is, cost-effectiveness analyses reveal findings that maximize the average outcome but are indifferent to the statistical distribution of these outcomes. Hence, it is unethical to control medical costs with bureaucratic mechanisms [22].

As medical economic evaluations are relatively specific to individual health care systems, translation of the results of one economic study to different health care systems can be problematic [15]. Tsuchiya et al. performed cost-effectiveness analyses of consolidation therapy with Pemetrexed for NSCLC in Japan [23]. Their data indicated difficulties in use of consolidation therapy as the standard of care for Japanese patients who are covered by general medical insurance. In contrast, a US study revealed consolidation therapy to be cost-effective. We calculated the direct costs of molecular-targeting drugs in the Japanese medical treatment fee system and devised a simple, reliable, and quantitative method, allowing physicians to re-calculate and estimate cost-effectiveness using drug costs that are specific to local health care systems.

The lack of QALY analyses is a limitation of the present study. However, the reliability of QALY estimates are limited by methodological variations in measurements of utilities [24], and how well the QALY system actually reflects patient preferences is still debated [25].

Another limitation of this study is that we only calculated molecular-targeting drug costs and did not include other direct medical costs such as premedication and supportive therapy, direct non-medical costs such as transportation, and indirect costs such as work loss. However, use of molecular-targeting drugs could add costs associated with adverse events.

## Conclusions

We have devised a simple and reliable method for estimating cost-effectiveness of novel chemotherapeutic agents and revealed poor cost-effectiveness of molecular-targeting drugs in Japan. Patient drug selections that are made with consideration of cost-effectiveness will save limited health care resources. Considering associated ethical and moral issues, economic endpoints for new drug approvals are required in Japan.

## Abbreviations

ICER: Incremental cost-effectiveness ratio; LYG: Life-year gained; NSCLC: Non-small-cell lung cancer; RCC: Renal cell carcinoma; HCC: Hepatocellular carcinoma; OS: Overall survival; PFS: Progression-free survival; BSC: Best supportive care; QALY: Quality-adjusted life year; CIRT: Carbon ion radiotherapy

## Competing interests

The authors declare that they have no competing interests.

## Authors’ contributions

TE designed the conception, collected and analyzed data, and drafted the manuscript. TO and TN helped to draft the manuscript. All authors read and approved the final manuscript.
